# Accuracy of clinical pallor in the diagnosis of anaemia in children: a meta-analysis

**DOI:** 10.1186/1471-2431-5-46

**Published:** 2005-12-08

**Authors:** Juan P Chalco, Luis Huicho, Carlos Alamo, Nilton Y Carreazo, Carlos A Bada

**Affiliations:** 1 Instituto de Salud del Niño, Lima, LI 05, Perú; 2 Universidad Peruana Cayetano Heredia, Lima, LI 05, Perú; 3 Universidad Nacional Mayor de San Marcos, Lima, LI 05, Perú; 4 Universidad San Martin de Porres, Lima, LI 05, Perú; 5 Hospital de Emergencias Pediátricas, Lima, LI 05 Perú

## Abstract

**Background:**

Anaemia is highly prevalent in children of developing countries. It is associated with impaired physical growth and mental development. Palmar pallor is recommended at primary level for diagnosing it, on the basis of few studies. The objective of the study was to systematically assess the accuracy of clinical signs in the diagnosis of anaemia in children.

**Methods:**

A systematic review on the accuracy of clinical signs of anaemia in children. We performed an Internet search in various databases and an additional reference tracking. Studies had to be on performance of clinical signs in the diagnosis of anaemia, using haemoglobin as the gold standard. We calculated pooled diagnostic likelihood ratios (LR's) and odds ratios (DOR's) for each clinical sign at different haemoglobin thresholds.

**Results:**

Eleven articles met the inclusion criteria. Most studies were performed in Africa, in children underfive. Chi-square test for proportions and Cochran Q for DOR's and for LR's showed heterogeneity. Type of observer and haemoglobin technique influenced the results. Pooling was done using the random effects model. Pooled DOR at haemoglobin <11 g/dL was 4.3 (95% CI 2.6–7.2) for palmar pallor, 3.7 (2.3–5.9) for conjunctival pallor, and 3.4 (1.8–6.3) for nailbed pallor. DOR's and LR's were slightly better for nailbed pallor at all other haemoglobin thresholds. The accuracy did not vary substantially after excluding outliers.

**Conclusion:**

This meta-analysis did not document a highly accurate clinical sign of anaemia. In view of poor performance of clinical signs, universal iron supplementation may be an adequate control strategy in high prevalence areas. Further well-designed studies are needed in settings other than Africa. They should assess inter-observer variation, performance of combined clinical signs, phenotypic differences, and different degrees of anaemia.

## Background

The global prevalence of anaemia is estimated in 2 billion people, that is, in about 30% of the worldwide population[1]. An even larger number of people present iron deficiency [1]. Every 9 of 10 persons affected of anaemia live in developing countries [2]. Anaemia prevalence in Latin America is 46% in children [3], with differences within countries. In Peru and Chile it is 50% and 8%, respectively [4,5].

Anaemia is related to impaired physical growth and mental development [6]. It is also associated to a higher risk of infant and child mortality, particularly when it co-exists with malnutrition and other risk factors [7].

It is therefore important to make a timely and accurate diagnosis and initiate an early intervention to reduce the negative impact of anaemia. The laboratory diagnosis of anaemia through any of several techniques is not widely available and its cost is often unaffordable in poor areas of the world. This stimulated several studies to assess the accuracy of clinical signs for screening of anaemia.

The Integrated Management of Childhood Illness (IMCI) strategy developed by the World Health Organization recommends the use of palmar pallor as the initial screening tool [8]. This recommendation is based mainly on the interpretation of results of studies performed in the Gambia [9], Kenya [10], and Malawi [11]. None of these studies showed in fact a clear superiority of palmar pallor. Only the Kenya study showed that palmar pallor performed better than conjunctival pallor when used by health workers but not by study physicians [10]. One of them used packed red cells volume as the gold standard [9]. Packed red cells volume is a controversial gold standard for anaemia, as it varies with different physiologic and pathologic conditions such as hydration status, and its correlation with haemoglobin is not optimal [12].

Thus we were prompted to perform a systematic review to assess the accuracy of clinical pallor in the diagnosis of anaemia. The specific objective of the study was to answer the question of whether there is a clinical sign that best predicts the presence or absence of anaemia in children. The signs most frequently assessed in primary studies are conjunctival, palmar and nailbed pallor. The review did not include respiratory and cardiovascular signs as they are unspecific for anaemia and are furthermore related to severe anaemia with haemodynamic repercussion.

## Methods

The review was aimed to include all studies performed in children aged 0 through 18 years old fulfilling pre-established inclusion criteria.

### Inclusion criteria

1. Studies on individual or combined accuracy of conjunctival, palmar or conjunctival pallor in the clinical diagnosis of anaemia.

2. Studies performed in children 0 through 18 years old.

3. Original articles. Review articles and letters to editors were not considered, except when they had enough information to assess the diagnostic performance of clinical signs of anaemia.

4. Prospective or retrospective studies performed in outpatient or inpatient children.

5. Articles with enough information to assess the diagnostic performance of clinical signs of anaemia, namely sensitivity, specificity, likelihood ratios and predictive values.

6. Studies in which haemoglobin was used as the gold standard.

### Exclusion criteria

1. Studies not related to assessment of clinical signs in the diagnosis of anaemia.

2. Studies with insufficient information for deriving the diagnostic performance of clinical signs.

3. Studies in which it was not used a gold standard or those in which haemoglobin was not the gold standard

### Search strategies

Two independent reviewers (JPC, CA) made an Internet search of the literature. The databases searched were the National Library of Medicine database from 1966 through January, 2002 and EMBASE from 1986 through January, 2002. In addition we searched the American and Caribbean Health Sciences Literature (Literatura Americana y del Caribe en Ciencias de la Salud, LILACS) database from 1986 through February, 2002 and the African Health Anthology database from 1924 through July, 2002. This search was combined with a manual tracking of articles deemed relevant and found in the references section of primary and qualitative review articles. Details of the key words used are presented as an appendix [See Additional File 1].

The abstracts of the primarily identified articles were read by the same two independent reviewers to assess whether they were related to the clinical diagnosis of anaemia. Those deemed to be relevant were then retrieved and read as full papers. Any discrepancy between the reviewers was solved by consensus.

### Methodological quality of primary studies

We assessed the methodological quality of primary studies according to modified published recommendations [13]. The quality score was derived by ascribing 2 points for each of the major criteria related to systematic and blind application of clinical signs and gold standard to all patients, and 1 point for each of the remaining criteria. The maximum possible score was 16 and the minimum was 0. The final validity rating was reached by consensus. The quality criteria details are presented as an appendix [see Additional File 2].

### Methods for calculating the diagnostic performance of index tests

Table 2 × 2 were reconstructed from the original data. Sensitivity, specificity, predictive values, and likelihood ratios with their corresponding 95% CIs were calculated for each primary study. Calculations were performed separately for each clinical sign and by different haemoglobin thresholds used in the primary studies. Whenever the 2 × 2 tables contained a 0 cell, 0.5 was added to all cells to avoid undefined results.

The diagnostic odds ratio (DOR) of each primary study was calculated according to the following formula [14]:

DOR = [Sensitivity/(1-sensitivity]/[(1-specificty)/specificity]

The DOR represents the ratio of the odds of a positive test result in subjects with the disease to the odds of a positive test result in subjects without the disease. A DOR of 1 means that the test has no discriminative power. When the DOR is more than one, the odds of a positive test result are higher in the diseased group.

### Methods of homogeneity assessment

Studies were analyzed separately for homogeneity of results by clinical sign and by haemoglobin threshold through chi-square test for proportions (sensitivity and specificity), through Cochran Q for LR's and DOR's [15] and through DOR graphic plotting of individual studies, along with their 95% CI graphs [16].

### Mathematical pooling

Pooled proportions (sensitivity and specificity) were calculated through the weighted averages taking into account the sample size of each study. Likewise, DOR's and LR's were pooled. The Mantel-Haenszel fixed effects model was planned to use if the studies were homogeneous for the diagnostic performance indexes and the DerSimonian Laird random effect model if they showed heterogeneity [17]. The 95% CIs were also calculated for all the pooled diagnostic indexes [16]. LR's and DOR's were recalculated after outlier's exclusion.

Diagnostic performance and 95% CIs of individual studies, homogeneity assessment, mathematical pooling and weighing were performed through the use of Metadisc software version Beta 1.1.1 [18].

### Exploration of heterogeneity

Potential sources of heterogeneity on diagnostic performance were assessed through Metaregression [18]. Pre-specified potential influential covariates included clinical setting (outpatients or inpatients), continent of study (Africa, Asia, Latin America), age group (children up to 5 years old, children older than 5 years old), technique of haemoglobin measurement (Hemocue^®^, spectrophotometry, Coulter^®^), whether or not the study setting was endemic for malaria or for intestinal worms, type of observer (physician, nurse, technician, parents), and methodological quality score (continuous variable). For each haemoglobin threshold category and for each test, multivariate metaregression was run including the above signaled covariates to assess whether any of them showed a significant influence on lnDOR. The metaregression was weighted by study size and the threshold effect was not considered, as there were not additional cutoff points within each pre-specified haemoglobin threshold.

### Post-test probabilities of anaemia

To graphically illustrate the relative usefulness of each particular clinical sign of anaemia at each haemoglobin threshold, different pre-test probability values were plotted against post-test probability values for both positive (LR+) and negative (LR-) results, before and after outlier's exclusion. The post-test probability for a disorder is another way to assess the value of a diagnostic test. It represents the chances that your patient has a disease. It incorporates information about the disease prevalence, the patient pool, and specific patient risk factors (pre-test probabilities) and information about the diagnostic test itself (the LR). The LR is used to assess how good a diagnostic test is and to help in selecting an appropriate diagnostic test(s) or sequence of tests. The LRs have advantages over sensitivity and specificity because they are less likely to change with the prevalence of the disorder, they can be calculated for several levels of the symptom/sign or test, they can be used to combine the results of multiple diagnostic test and they can be used to calculate the post-test probability for a target disorder. Post-test probabilities can be calculated for different clinical scenarios or settings with various possible pre-test probabilities (disease prevalence), using positive (LR+) and negative (LR-) results for the interest tests.

## Results

Adapted QUORUM statement checklist and flow diagram of the study are included as an appendix [see Additional File 3].

### Literature search

The number of primarily found articles was 225. Two hundred and two papers were excluded after abstract reading because they were nor relevant to the study objective. Twelve studies were excluded after reading them as full papers, because they were not performed in children (8 studies) [19-26], did not use haemoglobin as reference test (1) [27], did not assess individual signs of anaemia (1) [28], did not present separately results for children (1) [29], or did not perform clinical assessment of pallor (1) [30]. Finally, eleven articles were included in the meta-analysis [10,11,31-39].

All the studies we found had been performed in developing countries, mostly in children underfive. Eight were performed in Africa[10,11,31,35-39], one in Pakistan[32], one in Bangladesh and Uganda [34] and one in Brazil [33]. The Uganda component of one study was excluded as it used packed red cells volume as gold standard [34].

Most studies reported their results using pre-specified thresholds. All used one or more of the following haemoglobin categories: <11 g/dL, <8 g/dL, 7 g/dL, and < 5 g/dL. Only one study reported the results for 7 thresholds [36]. In this case, we re-constructed the results in the above noted 4 categories to allow the comparison of results with the other primary studies.

Table 1 summarizes main characteristics of primary studies, including the scores of methodological quality. There were studies that evaluated more than one sub-group of subjects and such results are shown separately.

**Table 1:** Summary of primary studies characteristics

**Author**	**Year**	**Country**	**Location**	**Ages**	**Number**	**Setting**	**Pallor**	**Haemoglobin cut-off assessed (g/dL)**
Wurapa FK^31^	1986	Zambia	Rural	< 4 y	12	Outpatient	C	<11
Thaver IH^32^	1994	Pakistan	Urban	6 m-5 y	947	Outpatient	C,N,P,T	<11
Luby SP^11^	1995	Malawi	Rural	< 6 y	1104	Outpatient	C,N,P,T	<11, <8, <5
Sdepanian VL^33^	1996	Brazil	Urban	6 m-5 y	143	Outpatient	C,G,N,P,T	<11
Kalter HD^34^	1997	Bangladesh	Urban	2 m-5 y	482	Emergency	C,P	<11, <8, <5
Zucker JR^10^	1997	Kenya	NS	2 m-5 y	1666	Outpatient	C,N,P,T	<8, <5
			NS	2 m-5 y	1048	Inpatient	C,N,P,T	<8, <5
Stoltzfus RJ^35^	1999	Tanzania	Urban	< 5 y^a^	613	Outpatient	C,N,P	<7,
			Urban	< 5 y^b^	537	Outpatient	C,N,P	<7
			Urban	> 5 y	3302	Outpatient	C,N,P	<7
Getaneh T^36^	2000	Ethiopia	Urban	2 m-5 y	574	Outpatient	C,N,P,T	<11, <8, <7, <5
Muhe L^37^	2000	Ethiopia	Rural	2 m-5 y	2540	Outpatient	C,N,P,T	<8, <5
Wamae CN^38^	2000	Kenya	Rural	2–4 y	574	Outpatient	P	<11
Desai MR ^29^	2002	Kenya	Rural	< 5 y	3782	Outpatient	C,G,N,P,T	<7, <5

**Observers**	**Kappa**	**Anaemia Prev.**	**Malaria area**	**Worm area**		**Haemoglobin Technique**		**Quality Score**

Physicians	No	16%	NS	NS		Coulter		12
Physicians	No	78%	NS	NS		Hemocue		12
Health workers	No	82%	NS	NS		Spectrophotometer		13
Paediatricians/Res.	No	41%	NS	NS		Coulter		11
Paediatricians	No	81%	No	NS		Hemocue		11
Physicians	No	59%	NS	NS		Hemocue		12
Physicians	No	91%	NS	NS		Hemocue		
Health workers	No	81%	NS	Yes		Hemocue		14
Health workers	No	52%	NS	Yes		Hemocue		
Health workers	No	32%	NS	Yes		Hemocue		
Nurses	Yes	46%	Yes	Yes		Hemocue		12
Physicians	Yes	61%	Yes	Ns		Hemocue		13
Health workers	No	61%	Yes	Yes		Spectrophotometer		12
Parents	No	66%	NS	NS		Hemocue		14

NS: Not stated, C: Conjunctiva, N: Nailbed, P: Palm, T: Tongue, G: General, Res.: residents, Prev.: prevalence. ^a ^and ^b ^are referred to cohorts studied in 1996 and 1997, respectively.

### Homogeneity assessment

**Figure 1:**
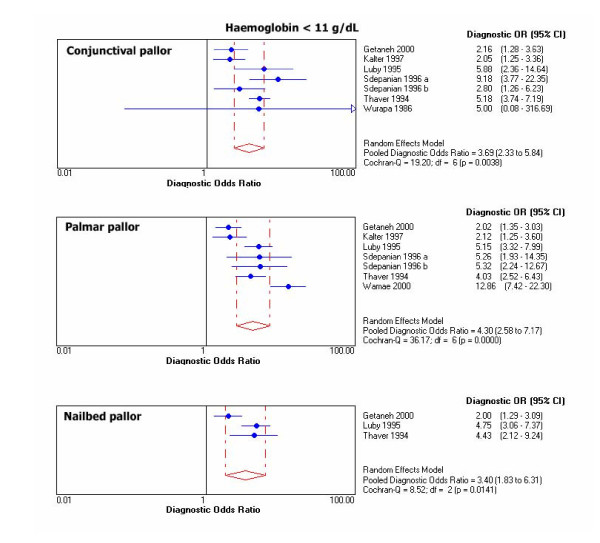
Individual and pooled DOR's at Hb <11 g/dL.

**Figure 2:**
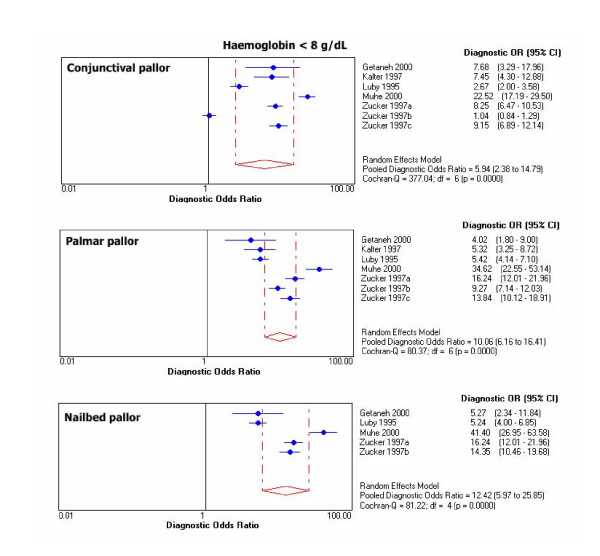
Individual and pooled DOR's at Hb <8 g/dL.

**Figure 3:**
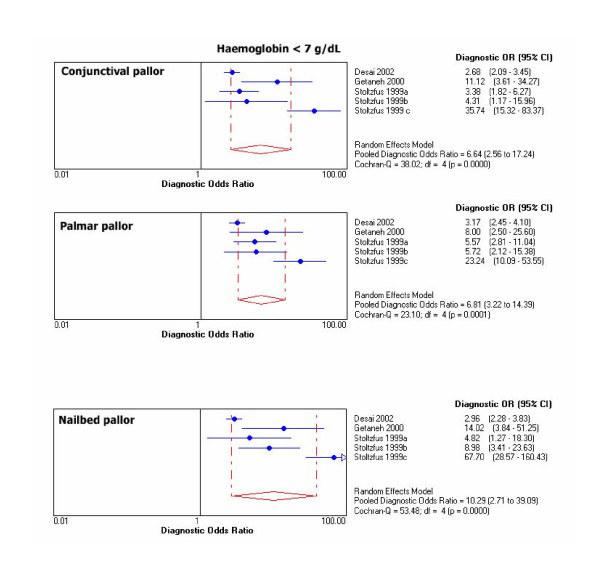
Individual and pooled DOR's at Hb <7 g/dL.

**Figure 4:**
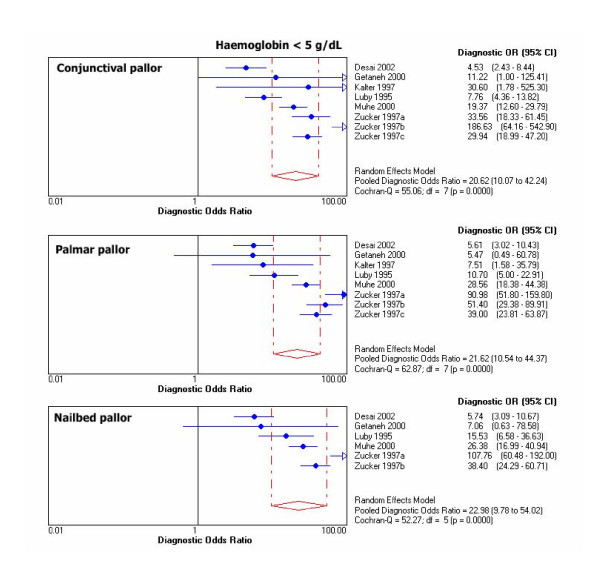
Individual and pooled DOR's at Hb <5 g/dL.

Chi-square test for proportions and Cochran Q for LR's and DOR's showed heterogeneity for results of primary studies within each threshold. For graphical display of the heterogeneity, 95% CIs for DOR's of individual studies are shown in Figure 1, 2, 3, 4.

Outliers at each haemoglobin category were identified through the DOR's graphs. Point estimates with confidence limits were plotted for each individual study. Those studies or results whose DOR's graphs were outside the 95% bounds of the pooled DOR were considered outliers. At haemoglobin <11 g/dL there was one outlier for palmar pallor [38]. At haemoglobin <8 g/dL there were 2 outliers for conjunctival pallor [10,37], one for nailbed [37], and one for palmar pallor [37]. At haemoglobin <5 g/dL there was 1 outlier for conjunctival pallor [39], 2 for palmar pallor [10,39], and one for nailbed pallor [39].

### Mathematical pooling

As diagnostic performance of primary tests showed heterogeneity, mathematical pooling for them was calculated using the DerSimonian Laird random effects model, to incorporate variation among studies. With this method the weighted average of LR's and DOR's logs are calculated. Tables 2, 3, 4, 5 show the pooled sensitivities, specificities, LR's, DOR's with their 95% CIs. Table 6 shows the pooled LR's and DOR's after exclusion of outliers.

**Table 2:** Pooled diagnostic performance markers at Hb <11 g/dL

**Clinical Pallor**	**Total N**	**Diseased**	**Sensitivity (95% IC)**	**Specificity (95% IC)**	**Likelihood Ratio+(CI, 95%)**	**Likelihood Ratio-(CI, 95%)**	**DOR (CI, 95%)**	AUC* SROC**
Conjunctiva	3195	2367	43.6(41.7–45.6)	81.4(78.6–83.9)	2.3(1.7–3.1)	0.7(0.5–0.9)	3.7(2.3–5.9)	0.7058
Palm	3885	2731	39.2(37.4–41.1)	86.7(84.6–88.5)	3.0(2.0–4.6)	0.7(0.6–0.8)	4.3(2.6–7.2)	0.7270
Nailbed	2534	1867	29.2(27.2–31.3)	88.5(85.8–90.7)	2.7(1.6–4.5)	0.8(0.7–0.9)	3.4(1.8–6.3)	0.6942

*AUC: area under the curve, **SROC: Summary receiver operating characteristics curve.

**Table 3:** Pooled diagnostic performance markers at Hb <8 g/dL

**Clinical Pallor**	**Total N**	**Diseased**	**Sensitivity (95% IC)**	**Specificity (95% IC)**	**Likelihood Ratio+(CI, 95%)**	**Likelihood Ratio-(CI, 95%)**	**DOR (CI, 95%)**	AUC* SROC**
Conjunctiva	8867	2711	70.8(69–72.5)	69(67.8–70.1)	2.5(1.5–4.1)	0.4(0.3–0.7)	5.9(2.4–14.8)	0.7687
Palm	8998	2713	80.9(79.4–82.3)	67.7(66.5–68.8)	2.7(2.3–3.0)	0.3(0.2–0.4)	10.1(6.2–16.4)	0.8274
Nailbed	6841	2043	79.7(77.9–81.4)	71.2(69.9–72.4)	3.0(2.5–3.6)	0.3(0.1–0.5)	12.4(5.9–25.9)	0.8477

*AUC: area under the curve, **SROC: summary receiver operating characteristics curve.

**Table 4:** Pooled diagnostic performance markers at Hb <7 g/dL

**Clinical Pallor**	**Total N**	**Diseased**	**Sensitivity (95% IC)**	**Specificity (95% IC)**	**Likelihood Ratio+(CI, 95%)**	**Likelihood Ratio-(CI, 95%)**	**DOR (CI, 95%)**	AUC* SROC**
Conjunctiva	8693	428	36.9(32.5–41.6)	88.7(88–89.4)	4.5(1.9–1.1)	0.8(0.6–0.9)	6.6(2.6–17.2)	0.7821
Palm	8726	429	35.7(31.3–40.3)	89.2(88.5–89.8)	4.2(2.3–7.7)	0.7(0.6–0.9)	6.8(3.2–14.4)	0.7851
Nailbed	8716	428	32.2(28–36.8)	90.8(90.1–91.4)	5.8(2.2–15.2)	0.6(0.5–0.9)	10.3(2.7–39.1)	0.8297

*AUC: area under the curve, **SROC: summary receiver operating characteristics curve

**Table 5:** Pooled diagnostic performance markers at Hb <5 g/dL

**Clinical Pallor**	**Total N**	**Diseased**	**Sensitivity (95% IC)**	**Specificity (95% IC)**	**Likelihood Ratio+(CI, 95%)**	**Likelihood Ratio-(CI, 95%)**	**DOR (CI, 95%)**	AUC* SROC**
Conjunctiva	12649	603	47.6(43.6–51.7)	88.1(87.5–88.7)	8.4(3.9–18.5)	0.6(0.5–0.7)	20.6(10.1–42.2)	0.8889
Palm	12780	603	56.6(52.5–60.5)	87.9(87.3–88.5)	7.7(3.1–19.0)	0.5(0.4–0.5)	21.6(10.5–44.4)	0.8922
Nailbed	10623	494	61.1(56.7–65.4)	87.7(87.1–88.4)	7.9(2.7–22.7)	0.4(0.4–0.5)	22.9(9.8–54.0)	0.8964

*AUC: area under the curve, **SROC: summary receiver operating characteristics curve.

**Table 6:** Pooled likelihood ratios and diagnostic odds ratios for index tests after excluding outliers

	**Hb <11 g/dL**			**Hb <8 g/dL**			**Hb <5 g/dL**		
	
**Clinical pallor**	**LR+**	**LR-**	**DOR**	**LR+**	**LR-**	**DOR**	**LR+**	**LR-**	**DOR**
**Conjunctiva**	---	---	---	2.6	0.4	6.4	7.1	0.5	19.7
	---	---	---	(2.2 – 3.0)	(0.2 – 0.7)	(3.7 – 11.0)	(2.8 – 17.6)	(0.4 – 0.7)	(11.5 – 33.5)
**Palmar**	2.6	0.8	3.5	2.7	0.3	8.3	6.9	0.5	23.8
	(1.8 – 3.6)	(0.7 – 0.8)	(2.3 – 5.1)	(2.2 – 3.2)	(0.2 – 0.5)	(5.5 – 12.7)	(2.3 – 20.4)	(0.4 – 0.6)	(13.4 – 42.3)
**Nailbed**	---	---	---	3.1	0.3	9.3	5.6	0.4	16.6
	---	---	---	(2.3 – 4.2)	(0.2 – 0.6)	(4.9 – 17.7)	(2.1 – 15.3)	(0.4 – 0.6)	(7.7 – 35.9)

Numbers in parenthesis denote 95% CIs. Dotted lines (---) denote absence of outliers. Hb <7 g/dL did not have outliers.

Pooled DOR at haemoglobin <11 g/dL was 4.3 (95% CI 2.6–7.2) for palmar pallor, 3.7 (2.3–5.9) for conjunctival pallor, and 3.4 (1.8–6.3) for nailbed pallor. For the same haemoglobin threshold, pooled LR+ was 3.0 (95% CI 2.0–4.6) for palmar pallor, 2.7 (1.6–4.5) for nailbed pallor, 2.3 (1.7–3.1) for conjunctival pallor. Also for haemoglobin <11 g/dL, pooled LR- was 0.7 (CI 95% 0.6–0.8) for palmar pallor, 0.7 (0.5–0.9) for conjunctival pallor, and 0.8 (0.7–0.9) for nailbed pallor. DOR's and LR's were slightly better for nailbed pallor at all other haemoglobin thresholds. The pooled diagnostic parameters did not vary substantially after excluding outliers, except that the modest DOR superiority for palmar pallor at haemoglobin <11 g/dL disappeared and improved over the other signs at haemoglobin <5 g/dL (Table 6).

### Exploration of heterogeneity

Method of haemoglobin measurement, type of examiner, continent, clinical setting (outpatients or inpatients) and quality score entered as covariates for most studies. Multivariate metaregression revealed that the only influential covariates on LnDOR for palmar pallor were type of observer (β = 3.16, p = 0.04; RDOR = 23.6, 95% CI = 1.05–531) and haemoglobin technique (β = -5.02, p = 0.03; RDOR = 0.01, 95% CI = 0.00–0.4) at haemoglobin <8 g/dL. There was not any other covariate significantly influencing on LnDOR for the other index clinical signs at any other haemoglobin threshold.

### Post-test probabilities of anaemia

**Figure 5:**
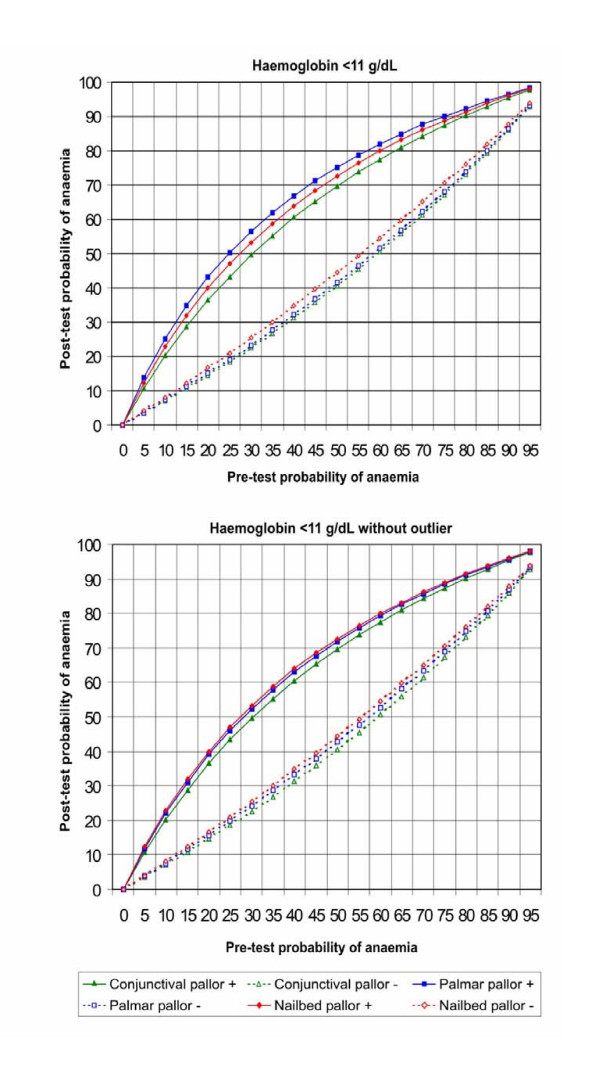
Post-test probabilities of positive and negative results of clinical signs of anaemia for different pre-test probabilities, at Hb<11 g/dL, with and without outliers.

**Figure 6:**
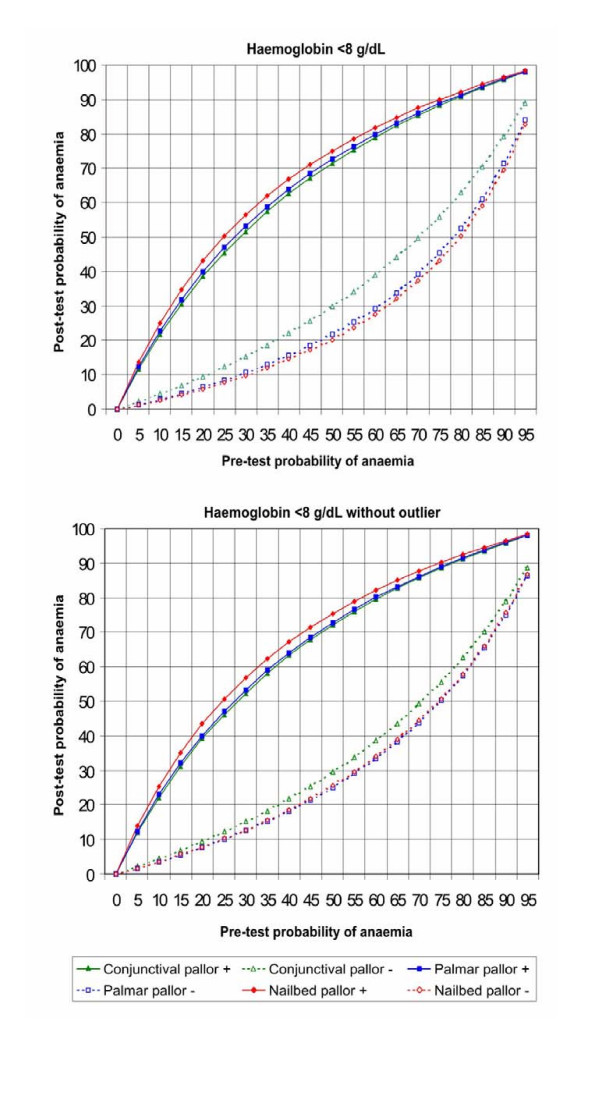
Post-test probabilities for positive and negative results of clinical signs of anaemia for different pre-test probabilities, at Hb<8 g/dL, with and without outliers.

**Figure 7:**
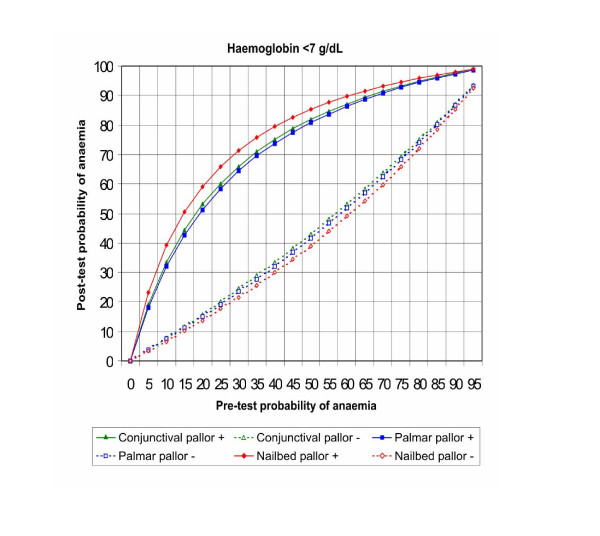
Post-test probabilities of positive and negative results of clinical signs of anaemia for different pre-test probabilities, at Hb<7 g/dL (no outliers were found).

**Figure 8:**
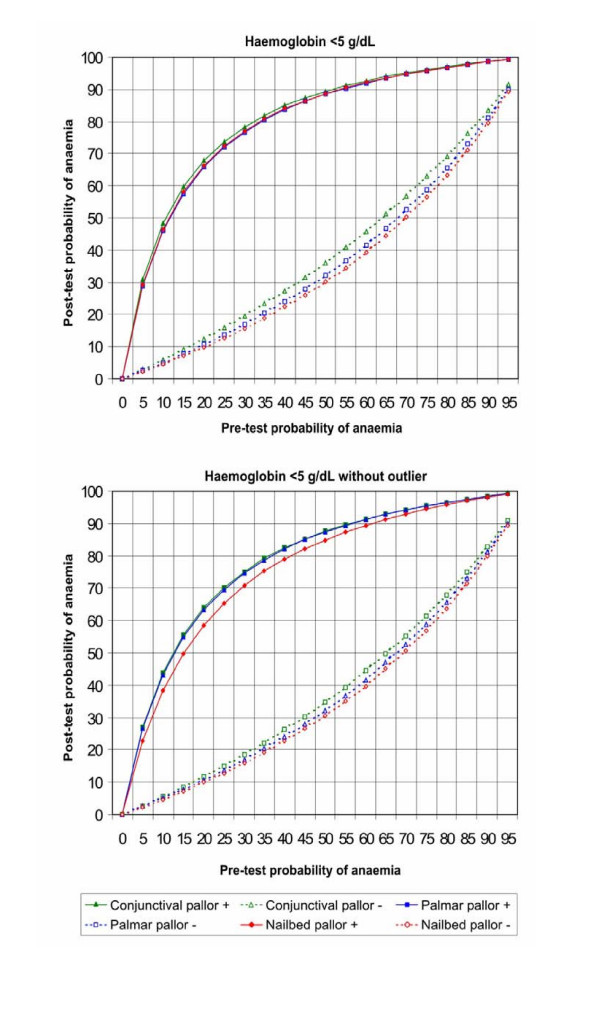
Post-test probabilities of positive and negative results of clinical signs of anaemia for different pre-test probabilities, at Hb<5 g/dL, with and without outliers.

Figures 5, 6, 7, 8 show the post-test probabilities for each particular sign at different haemoglobin thresholds before and after exclusion of outliers. Mild anaemia accounts for the greatest burden of disease in the world. Thus, considering different possible settings according to anaemia prevalence (pre-test probability), we illustrate here some post-test probabilities for positive and negative results of clinical signs of anaemia at haemoglobin <11 g/dL (Figure 5). For a clinical sign present and at 8% of anaemia prevalence, the post-test probability of disease increased to 21% for palmar pallor, to 19% for nailbed pallor, and to 17% for conjunctival pallor. For the same threshold and at 50% of anemia prevalence, the post-test probability increased to 75% for palmar pallor, to 73% for nailbed pallor, and to 70% for conjunctival pallor. And at 80% of anemia prevalence, the post-test probability of disease increased to 92% for palmar and nailbed pallor, and to 90% for conjunctival pallor. Like for DOR's and LR's, the discrete superiority of palmar pallor disappeared when outliers were excluded (Figure 5). At the same haemoglobin threshold (<11 g/dL), when a sign was absent, the post-test probability decrease was modest for any of the clinical signs of anaemia (Figure 5). The same trend was observed at other haemoglobin thresholds (Figures 6, 7, 8). Again, the exclusion of outliers did not change substantially the post-test probabilities.

## Discussion

We did not find a highly accurate clinical sign for diagnosing anaemia. Palmar pallor was modestly superior at haemoglobin less than 11 g/dL and nailbed was slightly superior at all other haemoglobin thresholds. After exclusion of outliers, nailbed pallor performed slightly better than the other signs, except at haemoglobin less than 5 g/dL, where palmar pallor improved somewhat over conjunctival and nailbed pallor.

Sensitivity of clinical signs ranged widely from 29.2% through 80.9% at different haemoglobin thresholds. Only palmar pallor showed 80.9% of sensitivity at haemoglobin less than 8 g/dL. And only nailbed pallor reached a 90.8% of specificity at haemoglobin less than 7 g/dL. This means that the rates of false positive and false negative results are unacceptably high for the clinical diagnosis of anaemia. The prevalence of asymptomatic anaemia in children may be as high as 87% in some areas of the world such south-eastern Tanzania [40]. Iron supplementation for all children in such a setting with a silent burden of anaemia has been suggested as a control strategy [40]. This can be associated with periodical deworming in tropical and subtropical countries [41].

A technical document supporting the use of palmar pallor as part of IMCI guidelines states that for detection of severe anaemia clinical signs should be as sensitive and specific as possible, to avoid missing referral for a potentially life-saving blood transfusion, and to avoid unnecessary referrals which would burden the families and the health facilities [8]. We did not find high values of sensitivity and specificity for any of the clinical signs of anaemia. Palmar pallor, the recommended sign, did not perform particularly better. The pooled sensitivities are higher and the pooled specificities lower than those for more severe anaemia. This is due to the fact that different studies did not assess necessarily the same haemoglobin cut-offs, which is shown in Table 1. By contrast, the diagnostic odds ratio, which constitutes a single test performance indicator, increases at lower haemoglobin cut-off values. This is one of the reasons we chose to include DOR's as another summary statistics for pooling accuracy of clinical signs in our study. In addition, DOR offers the advantage of overcoming the under-estimation of diagnostic accuracy that often occurs if one pools the results of primary studies just in terms of sensitivity and specificity.

We further explored whether the post-test probabilities of anaemia changed substantially when clinical signs were present or absent for different prevalences of the condition (pre-test probabilities). Pre-test probabilities of 50% and 8% represent the anaemia prevalence of Peru and Chile, respectively [4,5], and 80% is close to prevalence recently reported in southern Tanzania [40]. At haemoglobin less than 11 g/dL, post-test probabilities of anaemia did not show a substantial change in presence or absence of clinical signs, except in a scenario with 8% of prevalence. On an individual basis, a good clinical sign would lead correctly, when present, to prescribe iron and conversely, when absent, to withhold it. On the other side, an accurate diagnosis of severe anaemia should lead to a prompt referral for blood transfusion and additional interventions depending on the underlying causes of anaemia. However, at haemoglobin less than 5 g/dL, post-test probabilities of disease when a clinical sign was present increased up to 5 times only in a scenario with 8% of anaemia prevalence, but less than the double in scenarios with 50% and 80% of prevalence. The post-test probabilities decreased only slightly when a clinic sign was absent, at both haemoglobin less than 11 and 5 g/dL, irrespective of anaemia prevalence. Thus, this meta-analysis does not support the recommendation of taking a management decision on the basis of presence or absence of any of the clinical signs of anaemia assessed.

There are some limitations of primary studies included that may have influenced on the results of the meta-analysis. First, most studies did not assess inter-observer variation. Due to the subjective component in the appreciation of clinical pallor, it is important to quantify this factor. Second, most studies were performed in Africa, limiting their generalizability to other regions of the world due to phenotypic differences, varying anaemia prevalence and different causes such as malaria and intestinal parasitosis. For instance, it has been documented that a greater palmar pigmentation in Bangladesh was associated with a decreased sensitivity of palmar pallor [34]. In addition, high rates of blepharoconjunctivitis may obscure conjunctival pallor and also decrease its sensitivity [42]. Third, the diagnostic accuracy of combined signs was rarely performed [32,34]. Combining signs may increase the performance of clinical signs, even if such an evaluation may also increase in complexity. Trade-offs between combination of clinical signs and complexity of evaluation should be considered if combined signs display better diagnostic accuracy.

## Conclusion

We did not find a highly accurate clinical sign of anaemia. In view of poor performance of clinical signs, universal iron supplementation of children may be an adequate control strategy at public health level, particularly in high prevalence areas, as was recently suggested [40]. Further well-designed studies are needed for settings other than Africa.

They should assess inter-observer variation, performance of combined clinical signs, phenotypic differences and different degrees of anaemia.

## Competing interests

Luis Huicho is one of the principal investigators of the Multi-Country Evaluation of Integrated Management of Childhood Illness (IMCI), coordinated by the Department of Child and Adolescent Health of WHO and supported by the Bill and Melinda Gates Foundation and the US Agency for International Development. This project is aimed at evaluating the impact, cost and effectiveness of IMCI.

No other conflict of interest declared by any other author.

## Authors' contributions

J.PC took part in conception, design, data collection, management and analysis; LH in conception, design, data management and analysis; CA in data collection and analysis; NYC and CAB in data analysis. All authors contributed to interpretation of the data and writing of the report.

## Pre-publication history

The pre-publication history for this paper can be accessed here:



## Supplementary Material

Additional File 1 Key words used in the search. details of key words used in the search Click here for file

Additional File 2 Criteria for the assessment of the methodological quality of primary studies. Details of the quality criteria and scores ascribed for each of them Click here for file

Additional File 3 Adapted QUORUM statement checklist and flow diagram of the systematic review. It includes the completed QUORUM statement checklist and the flowchart Click here for file
