# Supplementary material for: Accuracy of clinical pallor in the diagnosis of anaemia in children: a meta-analysis
Source: BMC Pediatr. 2005 Dec 8;5:46. doi: 10.1186/1471-2431-5-46 (PMC1325025; doi:10.1186/1471-2431-5-46)
Supplement: Additional File 1 — Key words used in the search. details of key words used in the search [file 1471-2431-5-46-S1.doc]

**Additional File 1: Key words used in the search**

Clinical signs (Text word) OR signs (Text Word) OR pale (Text word) OR clinical pale (Text word) OR clinical exam (Text word) OR exam (Text word) OR clinical evaluation (Text word) OR evaluation (Text word) OR clinical assessment (Text word) OR assessment (Text word) OR pale conjunctival (Text word) OR conjunctiva (Text word) OR pale nailbed (Text word) OR nailbed (Text word) OR pale palm (Text word) OR palm (Text word) AND anaemia. "Limits 0 to 18 years" option was used for Medline.
