# Supplementary material for: Accuracy of clinical pallor in the diagnosis of anaemia in children: a meta-analysis
Source: BMC Pediatr. 2005 Dec 8;5:46. doi: 10.1186/1471-2431-5-46 (PMC1325025; doi:10.1186/1471-2431-5-46)
Supplement: Additional File 2 — Criteria for the assessment of the methodological quality of primary studies. Details of the quality criteria and scores ascribed for each of them [file 1471-2431-5-46-S2.doc]

**Additional File 2: Criteria for the assessment of the methodological quality of primary studies**

| 1. Sign(s) and "gold standard" applied systematically in all patients (Yes: 2; No: 0) | | | | |
| --- | --- | --- | --- | --- |
| 2. Sign(s) and "gold" standard applied blind and independently (Yes: 2, No: 0) | | | | |
| 3. Was the sign assessed in an appropriate spectrum of patients (like those in whom it would be used in practice)? (Yes: 2, No: 0) | | | | |
| 4.Technique of elicited sign stated (Yes: 1, No: 0) | |  | |  |
| 5.Threshold stated (Yes: 1, No: 0) |  | |  |  |
| 6.Patients Consecutive or Non-consecutive (Yes: 1, No: 0) | | | |  |
| 7.Study Prospective or Retrospective (Yes: 1, No: 0) | |  | |  |
| 8.Cohort symptomatic or not (Yes: 1, No; 0) | |  | |  |
| 9.Cohort description or not (Yes: 1, No: 0) | |  | |  |
| 10.Description of cohort assembly or not (Yes: 1, No: 0) | | | |  |
| 11. Age of cohort stated or not (Yes: 1, No: 0) | |  | |  |
| 12.Sex cohort stated or not (Yes: 1, No: 0) | |  | |  |
| 13. Comorbid conditions stated or not (Yes: 1, No: 0) | |  | |  |
