# Supplementary material for: Accuracy of clinical pallor in the diagnosis of anaemia in children: a meta-analysis
Source: BMC Pediatr. 2005 Dec 8;5:46. doi: 10.1186/1471-2431-5-46 (PMC1325025; doi:10.1186/1471-2431-5-46)
Supplement: Additional File 3 — Adapted QUORUM statement checklist and flow diagram of the systematic review. It includes the completed QUORUM statement checklist and the flowchart [file 1471-2431-5-46-S3.doc]

**Additional File 3: adapted QUORUM statement checklist and flow diagram of the systematic review**

**a. QUORUM statement checklist**

**_______________________________________________________________________________________________________________________________________________________________________________________**

**Heading Subheading Descriptor Reported? (Y/N) Page number**

**------------------- ----------------------------- ------------------------------------------------------------------------------------------------------------- --------------------------- -----------------------**

**Title**  Identify the report as a meta-analysis [or systematic review] of RCTs^26^ Yes 1

------------------- ---------------------------- ------------------------------------------------------------------------------------------------------------- --------------------------- -----------------------

**Abstract** Use a structured format^27^ Yes 2,3

**Describe**

Objectives The clinical question explicitly

Data sources The databases (ie, list) and other information sources

Review methods The selection criteria (ie, population, intervention, outcome, and study design);

methods for validity assessment, data abstraction, and study characteristics, and

quantitative data synthesis in sufficient detail to permit replication

Results Characteristics of the RCTs included and excluded; qualitative and quantitative

findings (ie, point estimates and confidence intervals); and subgroup analyses

Conclusion The main results

**------------------ ---------------------------- --------------------------------------------------------------------------------------------------------------- ---------------------------- -------------------------**

**Describe**

**------------------ ---------------------------- ---------------------------------------------------------------------------------------------------------------- ----------------------------- --------------------------**

**Introduction** The explicit clinical problem, biological rationale for the intervention, and rationale for review Yes 4,5

------------------ ----------------------------- ---------------------------------------------------------------------------------------------------------------- ------------------------------ --------------------------

**Methods**  Searching The information sources, in detail^28^ (eg, databases, registers, personal files, expert Yes 6,7

informants, agencies, hand-searching), and any restrictions (years considered, publication

status,^29^ language of publication^30,31^)

Selection The inclusion and exclusion criteria (defining population, intervention, principal Yes 6

outcomes, and study design^32^

Validity assessment The criteria and process used (eg, masked conditions, quality assessment, and their findings^33–36^) Yes 7,8

Data abstraction The process or processes used (eg, completed independently, in duplicate)^35,36^ Yes 6

Study characteristics The type of study design, participants’ characteristics, details of intervention, outcome Yes 7,8,9

definitions, &c,^37^ and how clinical heterogeneity was assessed

Quantitative data synthesis The principal measures of effect (eg, relative risk), method of combining results Yes 8,9

(statistical testing and confidence intervals), handling of missing data; how statistical

heterogeneity was assessed;^38^ a rationale for any a-priori sensitivity and subgroup analyses;

and any assessment of publication bias^39^

------------------ ----------------------------- --------------------------------------------------------------------------------------------------------------- ------------------------------ --------------------------

**Results** Trial flow Provide a meta-analysis profile summarising trial flow (see figure) Yes

Study characteristics Present descriptive data for each trial (eg, age, sample size, intervention, dose, duration,

follow-up period) Yes

Quantitative data synthesis Report agreement on the selection and validity assessment; present simple summary Yes

results (for each treatment group in each trial, for each primary outcome); present data

needed to calculate effect sizes and confidence intervals in intention-to-treat analyses

(eg 2X2 tables of counts, means and SDs, proportions)

--------------------- ---------------------------- ------------------------------------------------------------------------------------------------------------------- ------------------------------ -------------------------

**Discussion**  Summarise key findings; discuss clinical inferences based on internal and external validity; Yes

interpret the results in light of the totality of available evidence; describe potential

biases in the review process (eg, publication bias); and suggest a future research agenda

**________________________________________________________________________________________________________________________________________________________**

**b. QUOROM statement flow diagram**

Potentially relevant studies identified and screened for retrieval (n=225)

Studies retrieved for more detailed evaluation (n=23)

Potentially appropriate studies to be included in the meta-analysis (n=11)

Studies included in meta-analysis (n=11)

Studies with usable information, by outcome (n=11)

Studies excluded, with reasons (n= 202)

Studies excluded with reasons (n=12)

Studies excluded from meta-analysis, with reasons (n=0)

Studies withdrawn, by outcome, with reasons (n=0)
